# Supplementary material for: Structural basis of mammalian mucin processing by the human gut O-glycopeptidase OgpA from Akkermansia muciniphila
Source: Nat Commun. 2020 Sep 24;11:4844. doi: 10.1038/s41467-020-18696-y (PMC7518263; doi:10.1038/s41467-020-18696-y)
Supplement: Supplementary file 1 — Supplemental Information [file 41467_2020_18696_MOESM1_ESM.pdf]

SUPPLEMENTARY INFORMATION

Structural basis of mammalian mucin processing by the human gut *O*-glycopeptidase OgpA from *Akkermansia muciniphila*

Trastoy et al.

TABLE OF CONTENTS

1. **SUPPLEMENTARY TABLES**

Supplementary Table 1: Data Collection and Refinement Statistics.

Supplementary Table 2. Peptidases with known selectivity for heavily *O*-glycosylated proteins.

Supplementary Table 3: Structure and organization of *ogpA* gene.

2. **SUPPLEMENTARY RESULTS AND DISCUSSION**

3. **SUPPLEMENTARY FIGURES**

Supplementary Figure 1: Recombinant production of OgpA.

Supplementary Figure 2: Electron density map of the refined OgpA X-ray crystal structures.

Supplementary Figure 3: Structural homologues of OgpA.

Supplementary Figure 4: The catalytic mechanism of OgpA.

Supplementary Figure 5: Sequence alignment of OgpA with homologues found in the Verrucomicrobia and Bacteroidetes phyla.

Supplementary Figure 6: Substrate specificity of OgpA for *O*-glycopeptides I.

Supplementary Figure 7: Substrate specificity of OgpA for *O*-glycopeptides II.

Supplementary Figure 8: Genomic organization of *ogpA* gene in selected *A. muciniphila* strains.

4. **SUPPLEMENTARY REFERENCES**

1. SUPPLEMENTARY TABLES

Supplementary Table 1. Data collection and refinement statistics.

	$\mathbf{OgpA_{WT\text{-}SAD}}$ **(Zn)**	$\mathbf{OgpA_{WT1}}$	$\mathbf{OgpA_{WT2}}$	$\mathbf{OgpA_{H205A/E206A}}$**-GD-SUB**	$\mathbf{OgpA_{WT}}$**-GD-PRO**
PDB code		6Z2D	6Z2O	6Z2P	6Z2Q
Beamline	I24 (DLS)	I24 (DLS)	I24 (DLS)	BL13-XALOC	I03 (DLS)
Wavelength (Å)	1.28228	0.968820	1.170200	0.979181	0.976220
Resolution range (Å)	29.31-2.2	29.01 - 1.89	29.63 - 1.65	44.87 - 2.16	29.23 - 2.34
Space group	P 41 21 2	P 41 21 2	P 21 21 21	I 4	P 41 21 2
Unit cell	65.3, 65.3, 189.7, 90, 90, 90	64.9, 64.9, 187.4, 90, 90, 90	65.4, 70.2, 94.7, 90, 90, 90	126.9, 126.9, 65.5, 90, 90, 90	65.4, 65.4, 187.7, 90, 90, 90
Total reflections	372650 (7721)	805123 (62853)	670405 (53240)	111994 (9335)	224733 (16495)
Unique reflections	15013 (394)	32350 (2963)	52999 (5089)	27920 (2619)	17627 (1555)
Multiplicity	24.8 (19.6)	24.9 (21.2)	12.6 (10.5)	4.0 (3.6)	12.7 (10.6)
Completeness (%)	100 (97.8)	99.28 (93.14)	99.66 (97.03)	99.02 (93.63)	98.62 (87.13)
Mean I/sigma(I)	20.3 (28.1)	26.95 (3.78)	16.95 (3.29)	15.40 (1.42)	19.29 (2.91)
Wilson B-factor	20.1	26.11	23.23	51.51	35.98
R-merge	0.144 (0.094)	0.092 (0.84)	0.087 (0.57)	0.076 (0.77)	0.133 (0.96)
R-meas	0.15 (0.097)	0.094 (0.86)	0.091 (0.60)	0.087 (0.90)	0.139 (1.00)
CC1/2	0.99 (0.99)	1 (0.68)	0.99 (0.90)	0.99 (0.53)	0.999 (0.8)
CC*	0.99 (0.99)	1 (0.90)	0.99 (0.97)	0.99 (0.83)	1 (0.94)
Reflections used in refinement		32346 (2960)	52994 (5089)	27837 (2617)	17595 (1530)
Reflections used for R-free		1638 (131)	2729 (242)	1393 (131)	935 (82)
R-work		0.18 (0.25)	0.19 (0.22)	0.19 (0.31)	0.20 (0.26)
R-free		0.22 (0.30)	0.20 (0.30)	0.23 (0.35)	0.25 (0.29)
CC(work)		0.96 (0.77)	0.96 (0.89)	0.95 (0.73)	0.95 (0.81)
CC(free)		0.92 (0.74)	0.95 (0.75)	0.95 (0.74)	0.93 (0.71)
Number of non-hydrogen atoms		2971	3089	2913	2939
macromolecules		2784	2803	2849	2806
ligands		34	29	26	39
Protein residues		354	354	366	357
RMS(bonds)		0.007	0.006	0.009	0.013
RMS(angles)		0.84	0.83	0.98	1.34
Ramachandran favored (%)		98.30	97.71	97.24	97.73
Ramachandran allowed (%)		1.70	2.29	2.76	2.27
Ramachandran outliers (%)		0.00	0.00	0.00	0.00
Rotamer outliers (%)		0.00	0.34	0.34	0.70
Clashscore		0.90	2.33	1.97	4.71
Average B-factor		29.46	27.99	54.11	37.02
macromolecules		29.20	27.30	54.18	37.02
ligands		36.88	34.73	53.55	43.04
solvent		32.52	34.75	49.77	34.32

Statistics for the highest-resolution shell are shown in parentheses

Supplementary Table 2. Peptidases with known selectivity for heavily *O*-glycosylated proteins.

Name	Source	Substrate<br>Glycan Specificity	MEROPS Family	References	PDB codes
O-sialylglycoprotease *P. haemolytica* A1 protease	*Pasteurella haemolytica*	Glycophorin A	Former M22	1	
StcE (S/T*–X–S/T)[1]	*Escherichia coli*	MUC12, podocalyxin, CD43, PSGL-1, Syncam-1 and CD45<br>(core 1 and core2 *O*-glycans)	M66	2,3	3UJZ, 4DNY
Enhancins	*Trichoplusia ni*	Invertebral intestinal mucin (IIM)	M60	4	
SPATES protein		CD43, CD44, CD45, CD93, CD162 and CX3CL on human neutrophils and lymphocytes	S6	5	
BT4244	*Bacteroides thetaiotaomicron*	BSM<br>MUC-1<br>(GalNAc)	M60	6	5KD2<br>5KD5
IMPa	*Pseudomonas aeruginosa*	BSM<br>Bovine fetuin<br>Asialofetuin<br>(GalNAc, Galβ1–3GalNAc, Neu5cα2–3Galβ1–3GalNAc, Neu5cα2–6(Galβ1–3)GalNAc)	M88	6	5KDV<br>5KDW<br>5KDX
SslE	*E. coli*	BSM<br>Submaxillary gland mucin	M60	7	
ZmpB	*Clostridium perfringes*	BSM<br>Bovine fetuin<br>(Neu5cα2–6(Galβ1–3)GalNAc)	M60	6	5KDJ<br>5KDN<br>5KDS<br>5KDU

[1] StcE cleaves the peptide bond (*) at the position before the second serine or threonine and X is any amino acid. BSM: bovine submaxillary mucin

Supplementary Table 3. Structure and organization of *ogpA* gene.

Organism	Gene	Description	Prediction
Akkermansia muciniphila ATCC BAA-835	*AMUC_RS05935 or Amuc_1107*	ATP phosphoribosyltransferase	
	AMUC_RS12455 or Amuc_1108	Hypothetical protein	
	AMUC__RS11985 or Amuc_1109	Two-component sensor histidine kinase	
	AMUC_RS05955 or Amuc_1110	Response regulator transcription factor	
	AMUC_RS05960 or Amuc_1111	Dihydroxy-acid dehydratase (ilvD)	
	AMUC_RS05965 or Amuc_1114	Hypothetical protein	Autotransporter domain-containing protein
	AMUC_RS05970 or Amuc_1115	Integrase family protein	
	AMUC_RS05975 or Amuc_1116	Uracil-DNA glycosylase, phage SPO1 DNA polymerase-related protein	
	AMUC_RS05980 or Amuc_1117	2-iminoacetate synthase (ThiH) Biotin and thiamin synthesis associated protein	
	AMUC_RS05985 or Amuc_1118	Sulphatase	
	AMUC_RS11990 or Amuc_1119	**Hypothetical protein (OgpA)**	**Peptidase_M11**
	AMUC_RS05995 or Amuc_1120	Hypothetical protein	Putative GH95
	AMUC_RS06000 or Amuc_1121	Hypothetical protein	DUF2851 domain containing protein
	AMUC_RS11995 or Amuc_1122	STAS_anti-anti-sigma_factors; Sulphate Transporter and Anti-Sigma factor antagonist) domain of anti-anti-sigma factors, key regulators of anti-sigma factors by phosphorylation	
	AMUC_RS06010 or Amuc_1123	Hypothetical protein	Terpene cyclase/mutase family protein
	AMUC_RS06015 or Amuc_1124	Hypothetical protein	Terpene cyclase/mutase family protein
	AMUC_RS06020 or Amuc_1125	UDP-glucose 4-epimerase (GalE)	
	AMUC_RS06025 or Amuc_1126	Ribosomal RNA small subunit methyltransferase A (rsmA)	
	AMUC_RS06030 or Amuc_1127	Hypothetical protein	
	AMUC_RS06035 or Amuc_1128	Hypothetical protein	
	AMUC_RS12460 or Amuc_1129	Pseudo gene	
Akkermansia muciniphila CAG:154	*BN502_01331*	Miro domain protein	
	BN502_01332	Pyridoxal phosphate homeostasis protein or PLP Homeostasis	
	BN502_01333	Hypothetical protein	
	BN502_01334	Hypothetical protein	erfK/YbiS/YcfS/YnhG family protein
	BN502_01335	Hypothetical protein	Glycosyl transferase family 2
	BN502_01336	Hypothetical protein	
	BN502_01337	Hypothetical protein	
	BN502_01338	Non-specific serine/threonine protein kinase	
	BN502_01339	Hypothetical protein	Biotin and thiamin synthesis associated
	BN502_01340	Sulphatase	
	BN502_01341	**Hypothetical protein**	
	BN502_01342	Hypothetical protein	Putative GH95
	BN502_01343	Hypothetical protein	
	BN502_01344	Anti-sigma factor antagonist	
	BN502_01345	Hypothetical protein	Terpene cyclase/mutase family protein

	BN502_01346	Hypothetical protein	
	BN502_01347	Hypothetical protein	
	BN502_01348	UDP-glucose 4-epimerase	
	BN502_01349	Dimethyladenosine transferase	
	BN502_01350	Hypothetical protein	
	BN502_01351	Hypothetical protein	
Akkermansia sp.	*DDX86_00800*	Hypothetical protein	
	DDX86_00805	Hypothetical protein	ABC Transporter
	DDX86_00810	Molecular chaperone DnaJ	
	DDX86_00815	16S rRNA (uracil(1498)-N(3))-methyltransferase	
	DDX86_00820	NUDIX hydrolase	
	DDX86_00825	Anti-sigma factor antagonist	
	DDX86_00830	Hypothetical protein	
	DDX86_00835	Hypothetical protein	DUF2851 domain containing protein
	DDX86_00840	Hypothetical protein	Putative GH95
	DDX86_00845	Hypothetical protein	Putative GH95
	DDX86_00850	**Hypothetical protein**	
	DDX86_00855	Sulphatase	
	DDX86_00860	2-iminoacetate synthase (ThiH)	
	DDX86_00865	Hypothetical protein	Thioredoxin domain-containing protein
	DDX86_00870	Phosphomethylpyrimidine synthase	
	DDX86_00875	Hypothetical protein	
	DDX86_00880	Hypothetical protein	Peptide chain release factor 2
	DDX86_00885	Hypothetical protein	Peptide chain release factor 2
	DDX86_00890	tRNA (guanosine(37)-N1)-methyltransferase (TrmD)	
	DDX86_00895	Small basic protein	
	DDX86_00900	Pseudouridine synthase	
Akkermansia sp. CAG:344 WGS	*BN616_02297*	Hypothetical protein	
	BN616_02298	Hypothetical protein	TfoX_C domain-containing protein
	BN616_02299	Dimethyladenosine transferase	
	BN616_02300	UDP-glucose 4-epimerase	
	BN616_02301	Hypothetical protein	
	BN616_02302	Hypothetical protein	
	BN616_02303	Hypothetical protein	
	BN616_02304	Anti-sigma factor antagonist	
	BN616_02305	Hypothetical protein	DUF2851 domain containing protein
	BN616_02306	Hypothetical protein	Putative GH95
	BN616_02307	**Hypothetical protein**	
	BN616_02308	Sulphatase	
	BN616_02309	Hypothetical protein	Biotin and thiamin synthesis associated protein
	BN616_02310	Hypothetical protein	Phage SPO1 DNA polymerase-related protein
	BN616_02311	Integrase family protein	
	BN616_02312	Autotransporter barrel domain protein	
	BN616_02313	Hypothetical protein	
	BN616_02314	dihydroxy-acid dehydratase	
	BN616_02315	two component transcriptional regulator	
	BN616_02316	Histidine kinase	
	BN616_02317	Hypothetical protein	
Akkermansia sp. KLE1798	*HMPREF3039_02178*	Carbamoyl-phosphate synthase, small subunit	
	HMPREF3039_02179	GDP-mannose 4,6-dehydratase	
	HMPREF3039_02180	Hypothetical protein	NAD dependent epimerase/dehydratase family protein
	HMPREF3039_02181	Na+/H+ antiporter family protein	
	HMPREF3039_02182	Hypothetical protein	PA14 domain-containing protein
	HMPREF3039_02183	Phosphomannose isomerase type I	
	HMPREF3039_02184	N-acetylmuramoyl-L-alanine amidase	

	HMPREF3039_02185	Hypothetical protein	
	HMPREF3039_02186	Hypothetical protein	DNA-directed RNA polymerase subunit alpha
	HMPREF3039_02187	Hypothetical protein	Putative GH95
	HMPREF3039_02188	Hypothetical protein	
	HMPREF3039_02189	Arylsulfatase	
	HMPREF3039_02190	Hypothetical protein	
	HMPREF3039_02191	Hypothetical protein	Thiazole biosynthesis protein (ThiH)
	HMPREF3039_02192	Hypothetical protein	Uracil-DNA glycosylase
	HMPREF3039_02193	Hypothetical protein	Glycosyltransferase
	HMPREF3039_02194	Hypothetical protein	
	HMPREF3039_02195	Hypothetical protein	
	HMPREF3039_02196	RHS repeat-associated core domain protein	
	HMPREF3039_02197	Hypothetical protein	
	HMPREF3039_02198	Hypothetical protein	

2. SUPPLEMENTARY RESULTS AND DISCUSSION

The catalytic mechanism of OgpA.

We are now able to visualize two steps of the catalytic cycle of OgpA: (i) the binding of *O*-glycopeptide substrate to the active site of the enzyme ($OgpA_{H205A/E206A}$-GD-SUB), and (ii) the binding of the primed *O*-glycopeptide product before being released from the active site ($OgpA_{WT}$-GD-PRO) (Supplementary Fig. 3a). OgpA displays a conserved metal binding site HEXXHXXXXXH.[8] The proposed catalytic mechanism is similar to that described for thermolysin from *Bacillus thermoproteolyticus* and bovine carboxypeptidase A[9–11] decades ago, and more recently for matrix metalloproteases (MMPs).[12,13] It corresponds to a single-displacement that comprises the nucleophilic attack of a catalytic solvent molecule polarized by the general base/acid glutamate and the catalytic Zn^{2+} atom (Supplementary Fig. 3b).[9,11,13–17] The water molecule is part of the functional enzyme. It coordinates first with the metal atom, and it is polarized by the base/acid glutamate which enhances its nucleophlity. Once the peptide substrate is bound in the active cleft, the scissile carbonyl oxygen is polarized by the catalytic Zn^{2+} atom, thus the water molecule is able to attack the carbonyl oxygen of the peptide and simultaneusly transfer a proton to the general base glutamate. The nucleophilic attack produces a *gem*-diolate tetrahedral reaction intermediate that it is stabilized by the Zn^{2+} atom and neighboring residues. This intermediate leads to the formation of a double-product complex mediated by scissile bond cleavage and double proton tranfer to the new α-amino terminus. Finally, both products are released from the enzyme, most likely the nonprimed product first.

The Zn^{2+} atom coordinates with three histidines residues, H205, H209 and H215, and three water molecules in the $OgpA_{WT1}$ crystal form, and two water molecules and one ethylenglycol molecule in the $OgpA_{WT2}$ crystal form, adopting in both cases an octahedral geometry almost regular (Supplementary Fig. 3). Although, the lowest-energy ground-state coordination number of zinc bound to one acidic or two or more neutral protein ligands is 4,[15] the octahedral geometry has been observed in other unliganded metalloproteases.[12] The base/acid residue E206 is in close contact with the

catalytic water molecule in both crystal forms, $OgpA_{WT1}$ and OgA_{WT2} (2.9 Å and 2.5 Å, respectively). In addition, Y236 makes hydrogen bonds with the base/acid residue E206 and partially occupies the substrate binding site in the $OgpA_{WT2}$ crystal structure, while in $OgpA_{WT1}$ loop 9 adopts a different conformation and Y236 is positioned distant to E206 (Supplementary Fig. 3a). Due to the mutation of H205 by alanine, the Zn^{2+} cation is not present in the active site of $OgpA_{H205A/E206A}$-GD-SUB structure. The entrance of the substrate does not cause conformational changes in the loops that surround the binding pocket. However, the position of Y236 changes to make hydrogen bonds with the *O*-glycopeptide backbone. In the absence of the Zn^{2+} atom, the scissile carbonyl oxygen is making hydrogen bonds with Y318 and H215, at a distance that could be coordinated by the Zn^{2+} atom showed in the unliganded crystal structures (2.4 Å in $OgpA_{WT1}$ and $OgpA_{WT2,}$ respectively). In the $OgpA_{WT}$-GD-PRO crystal structure, the nonprimed peptide product is not present, supporting the notion that it is the first to leave the active site. In addition, the Zn^{2+} is tetracoordinated with an acetate molecule from the crystallization condition. The amino terminus of the GD-PRO interacts with E206 by hydrogen bonds and the rest of the interactions that maintain the primed peptide in the active site are mediated by the *O*-glycan and the protein.

3. SUPPLEMENTARY FIGURES

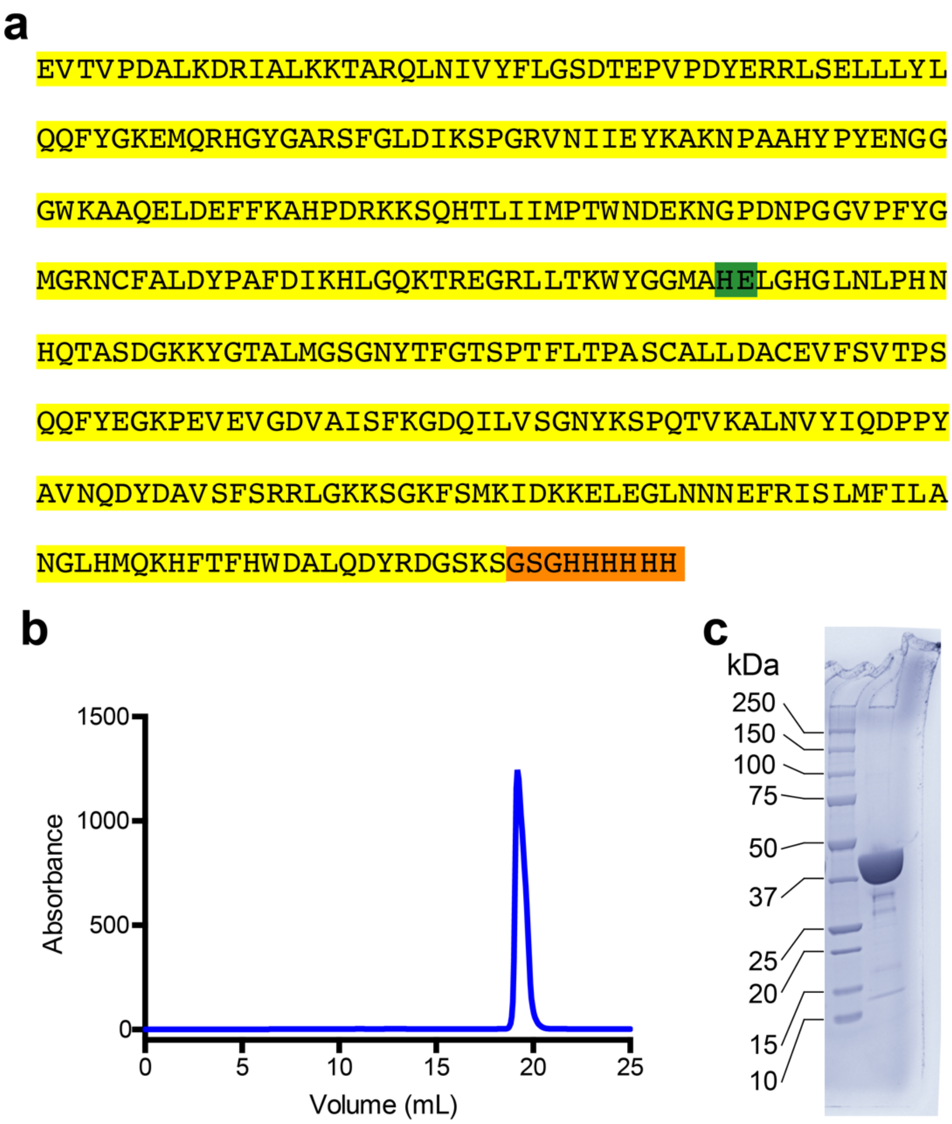

Supplementary Figure 1 | Recombinant production of OgpA. a The recombinant OgpA construct (residues 25-385) is highlighted in yellow. Additional residues are highlighted in orange. Catalytic residues are highlight in green. To obtain a catalytically inactive OgpA, H205 and E206 residues were replaced by alanine. **b** Superdex 200 Increase 10/300 GL profile showing purified monomeric OgpA. **c** SDS-PAGE showing purified OgpA. The sample was run in one gel.

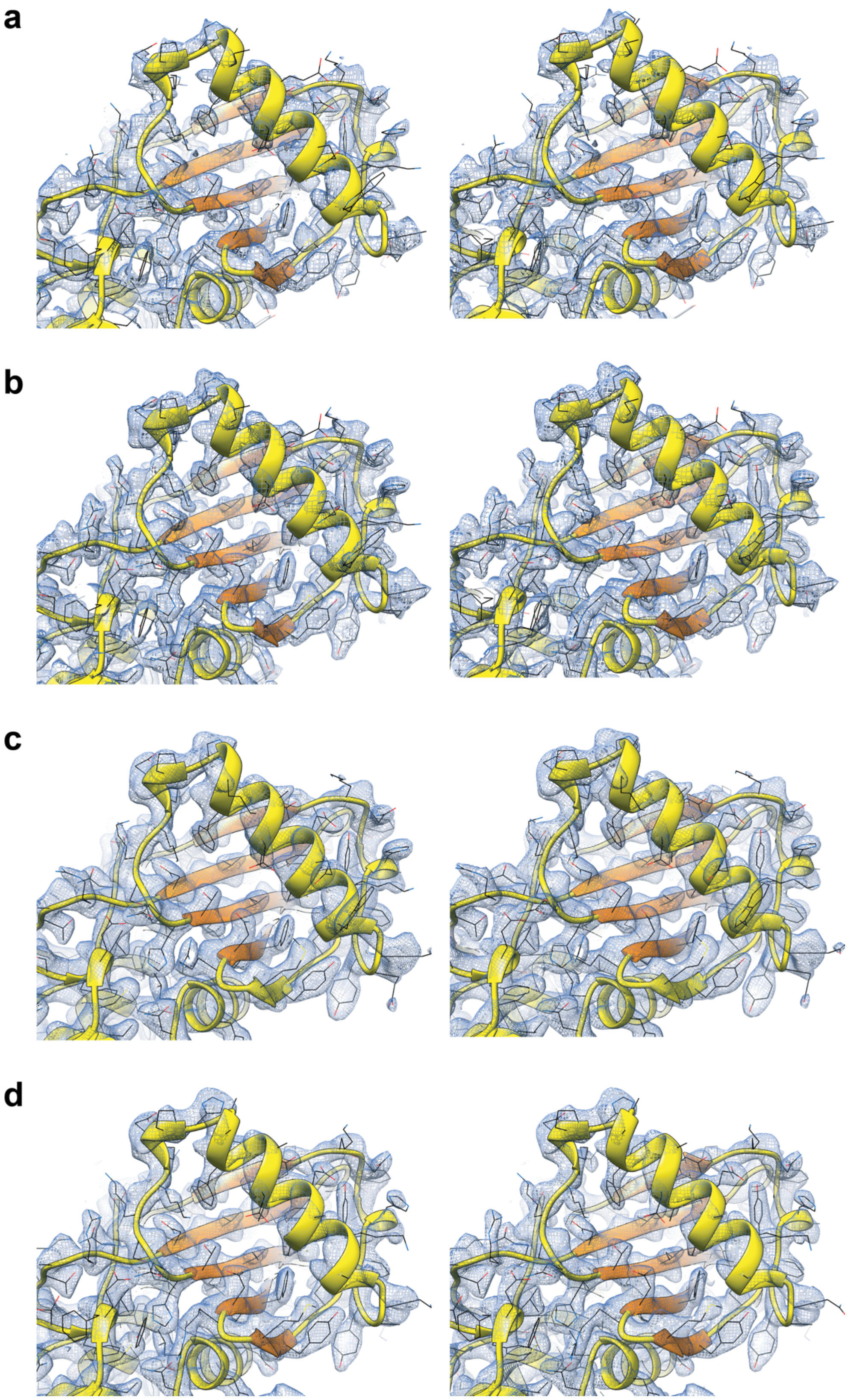

Supplementary Figure 2 | Electron density map of the refined OgpA X-ray crystal structures. Stereo view of the final electron density maps (2mFo-DFc contoured at 1σ) corresponding to the OgpA$_{WT1}$ (**a**), OgpA$_{WT2}$ (**b**), OgpA$_{H205A/E206A}$-GD-SUB **(c)**, OgpA$_{WT}$-GD-PRO **(d)** structures.

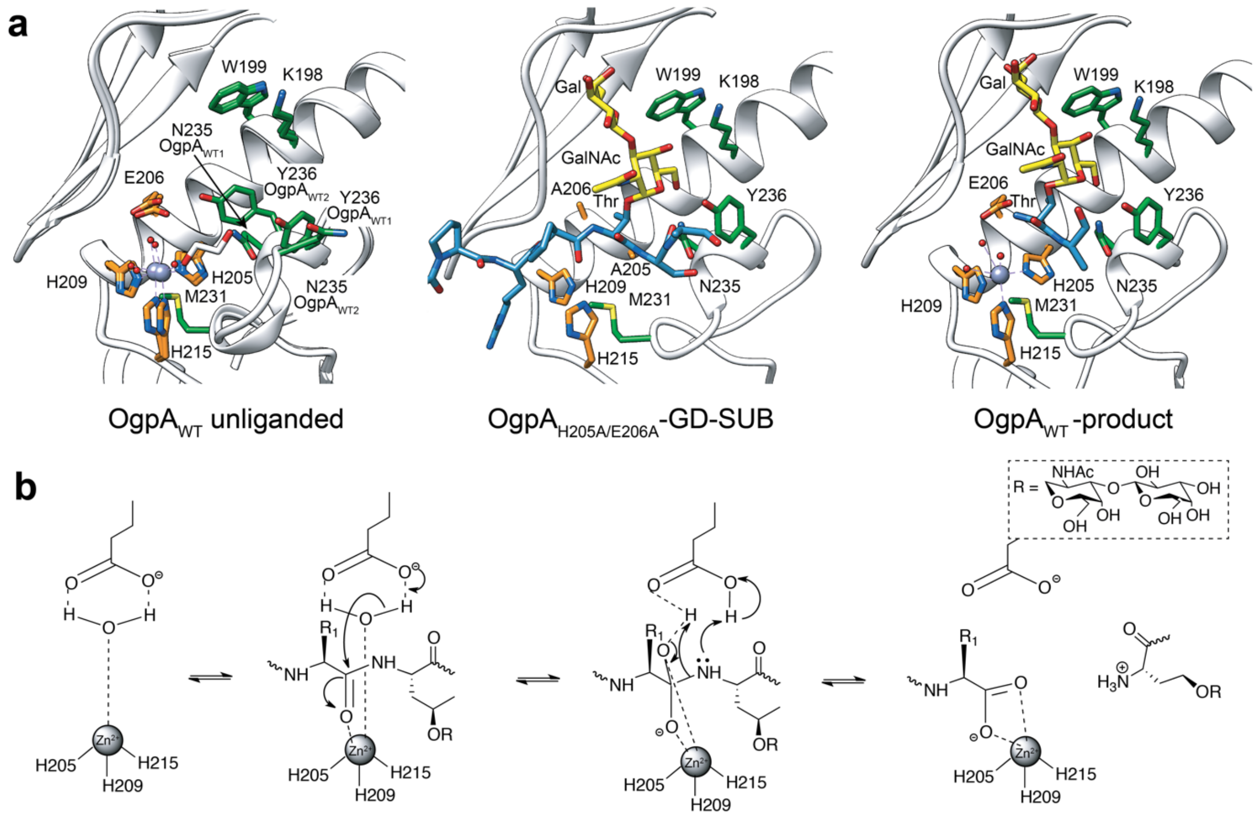

Supplementary Figure 3 | The catalytic mechanism of OgpA. a Catalytic site of the superimposed $OgpA_{WT1}$ and $OgpA_{WT2}$ unliganded (left), $OgpA_{H205A/E206A}$-GD-SUB (center) and $OgpA_{WT}$-GD-PRO (right) crystal structures. **b** Proposed catalytic mechanism for OgpA.

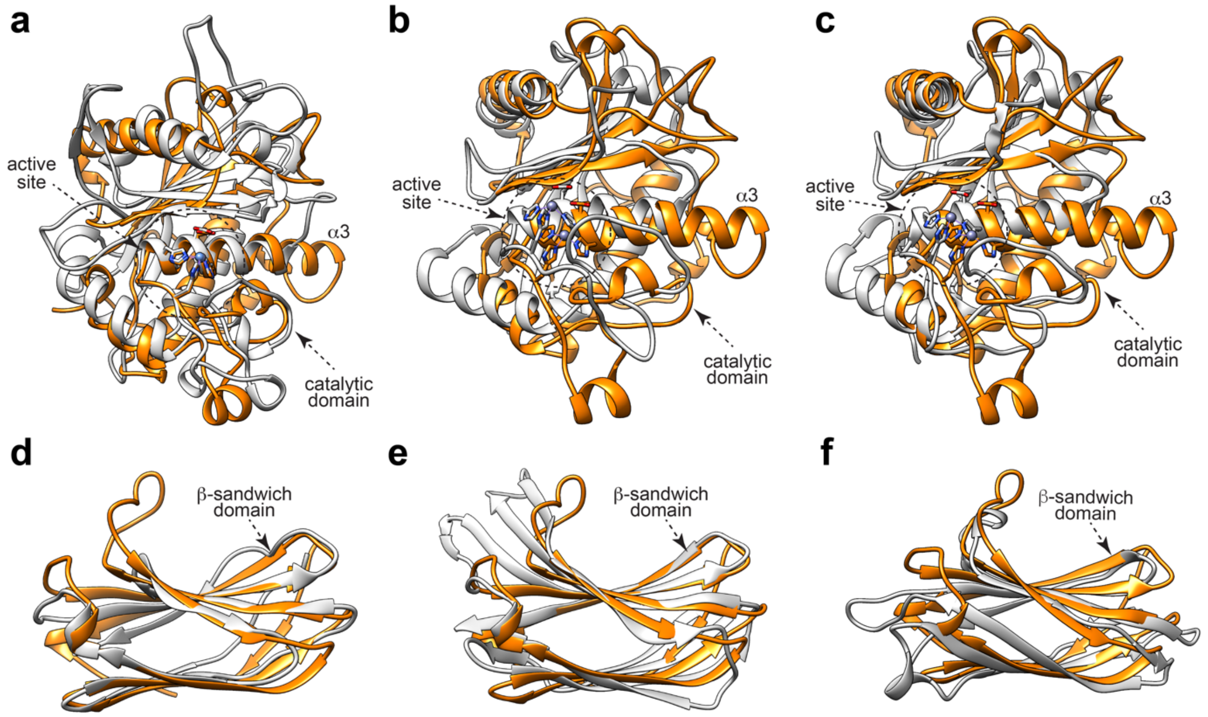

Supplementary Figure 4 | Structural homologues of OgpA. Structural superposition of the X-ray crystal structure of $OgpA_{WT1}$ (orange) and structural homologues (grey): **a** TNF-alpha converting enzyme (TACE; PDB code 3G42), **b** Atrolysin C from *C. atrox* (PDB code 1ATL), **c** *Ba*P1 from *B. asper* (PDB code 1ND1), **d** FnIII domain of SleM from *C. perfringens* (PDB code 5JIP), **e** FnIII domain of vacuolar protein sorting-associated protein 26A (VPS26A; PDB code 6H7W), **f** β-sandwich domain of glycoside hydrolase XacMan2A from *X. axonopodis* pv. citri (5DMY; PDB code).

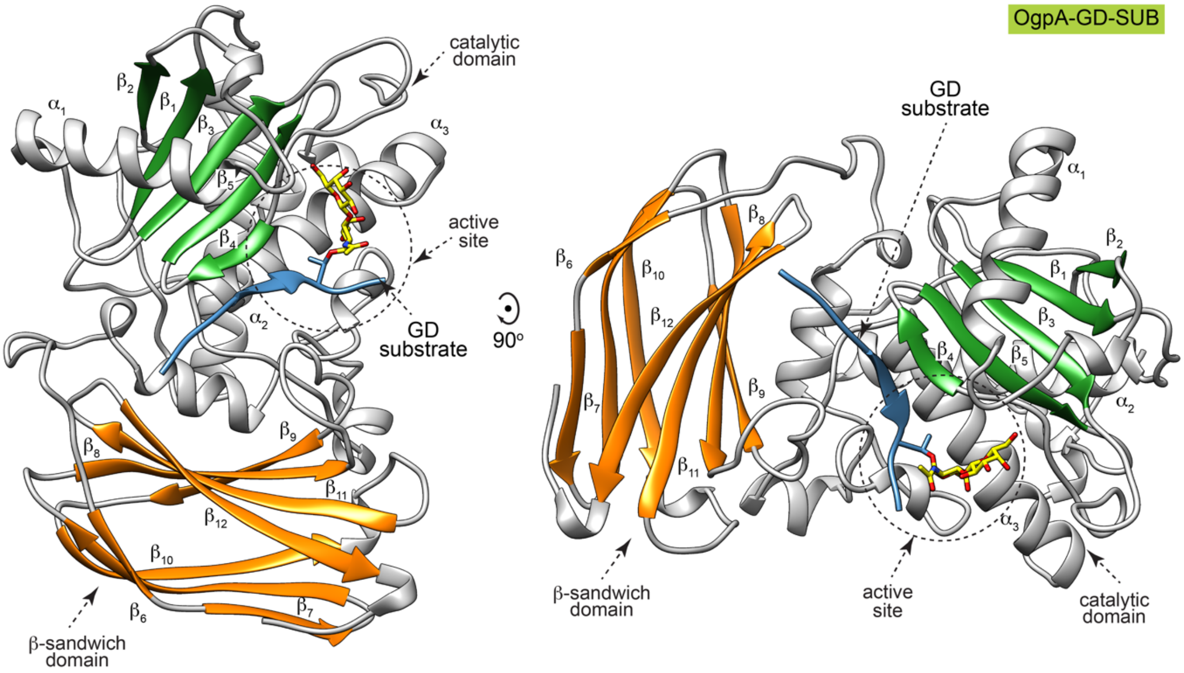

Supplementary Figure 5 | Cartoon representation showing the general fold and secondary structure organization of OgpA. Secondary structure elements are labelled. The central β-sheet of the catalytic domain and the β-sandwich are coloured in green and orange, respectively. The *O*-glycopeptide substrate is coloured in blue. It forms a parallel β-strand with respect to β2, β1, β3 and β5 comprised in the β-sheet of the catalytic domain. The disaccharide GalNAcGal is coloured in yellow.

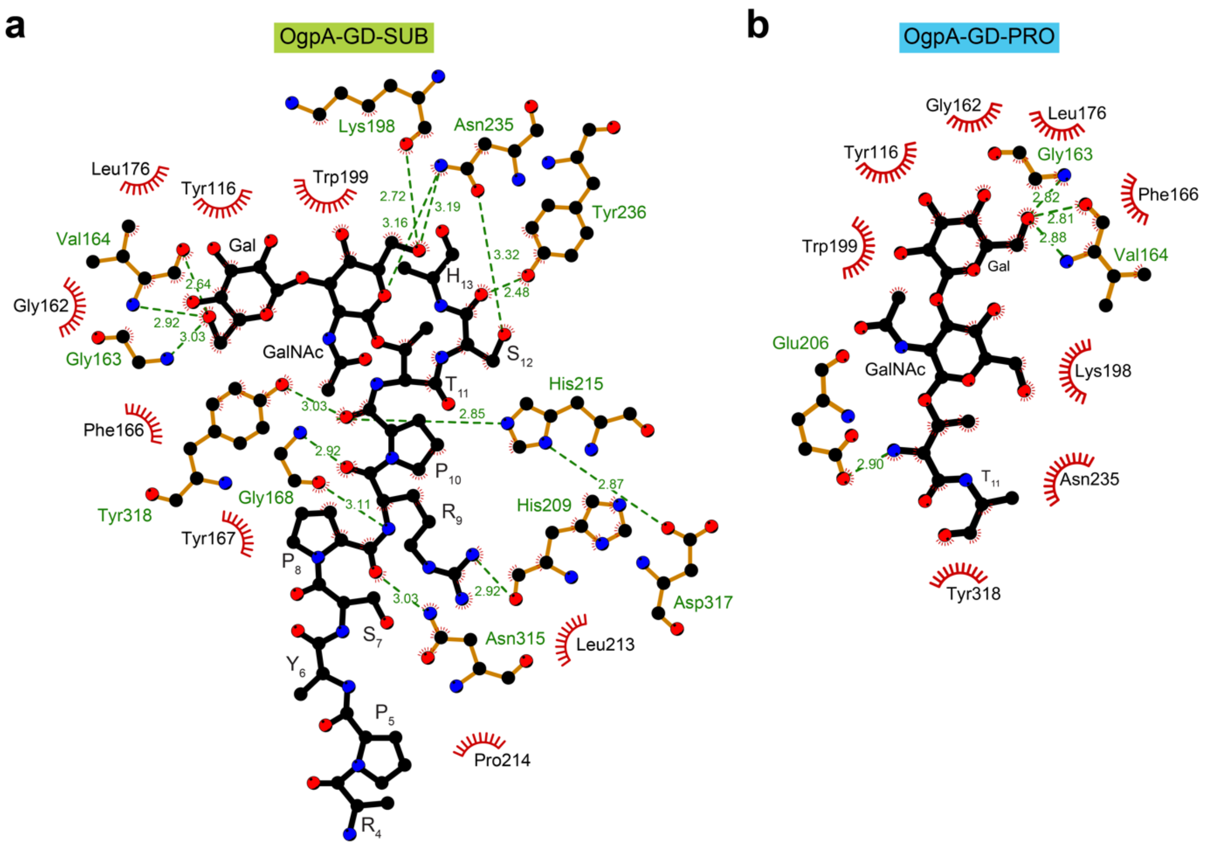

Supplementary Figure 6 | OgpA-SUB and OgpA-PRO full interaction profiles. Schematic diagrams of protein-ligand interactions for **a** $OgpA_{H205A/E206A}$-GD-SUB (PDB code 6Z2P) and **b** $OgpA_{WT}$-GD-PRO (PDB code 6Z2Q). The interactions shown are those mediated by hydrogen bonds (green) and by hydrophobic contacts (orange). Hydrogen bonds are indicated by dashed lines between the atoms involved, while hydrophobic contacts are represented by an arc with spokes radiating towards the ligand atoms they contact.

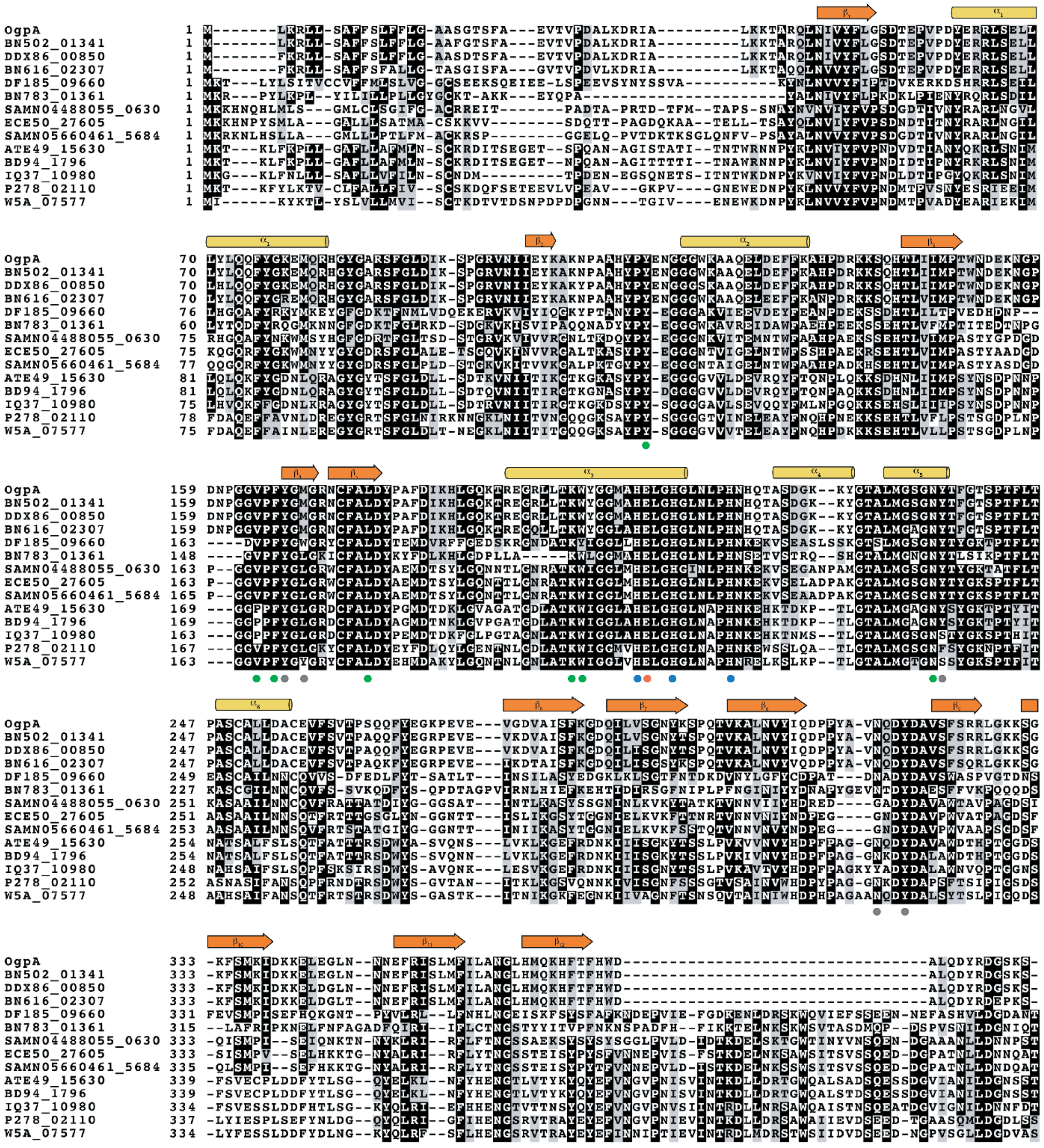

Supplementary Figure 7 | Sequence alignment of OgpA with homologues found in the Verrucomicrobia and Bacteroidetes phyla. Comparison of OgpA from *A. muciniphila* (B2UR60, Uniprot code), with BN502_01341 from *A. muciniphila* CAG:154 (R6J0R4, Uniprot code, Identity: 99 %), DDX86_00850 from *Akkermansia* sp. (A0A354E6N0, Uniprot code, Identity: 97 %), BN616_02307 from *Akkermansia* sp. *CAG:344* (R7DZB7, Uniprot code, Identity: 91%), DF185_09660 from *Marinifilum breve* (A0A2V3ZZK2, Uniprot code, Identity: 38 %), BN783_01361 from *Odoribacter* sp. *CAG:788* (R5PJX3, Uniprot code, Identity: 47%), SAMN04488055_0630 from *Chitinophaga niabensis* (A0A1N6DD36, Uniprot code, Identity: 41%), ECE50_27605 from *Chitinophaga* sp. *Mgbs1* (A0A3S1CWX3, Uniprot code, Identity: 43%), SAMN05660461_5684 from *Chitinophaga ginsengisegetis* (A0A1T5PAV1, Uniprot code, Identity: 44%), ATE49_15630 from *Elizabethkingia miricola* (A0A1A6C9I9, Uniprot code, Identity: 44%),

BD94_1796 from *Elizabethkingia anophelis* (A0A077EDG2, Uniprot code, Identity: 39%), IQ37_10980 from *Chryseobacterium piperi* (A0A086BCN5, Uniprot code, Identity: 41%), P278_02110 from *Zhouia amylolytica* (W2URL6, Uniprot code, Identity: 38%), W5A_07577 from *Imtechella halotolerans* (I0WF82, Uniprot code, Identity: 41%). The catalytic residue and the residues that coordinate the Zn atom are marked with red and blue dots, respectively. The residues that interact with the Gal-GalNAc glycan and the aminoacids of the GD substrate in the crystal structure of $OgpA_{H205A/E206A}$-GD-SUB are marked with green and grey dots, respectively.

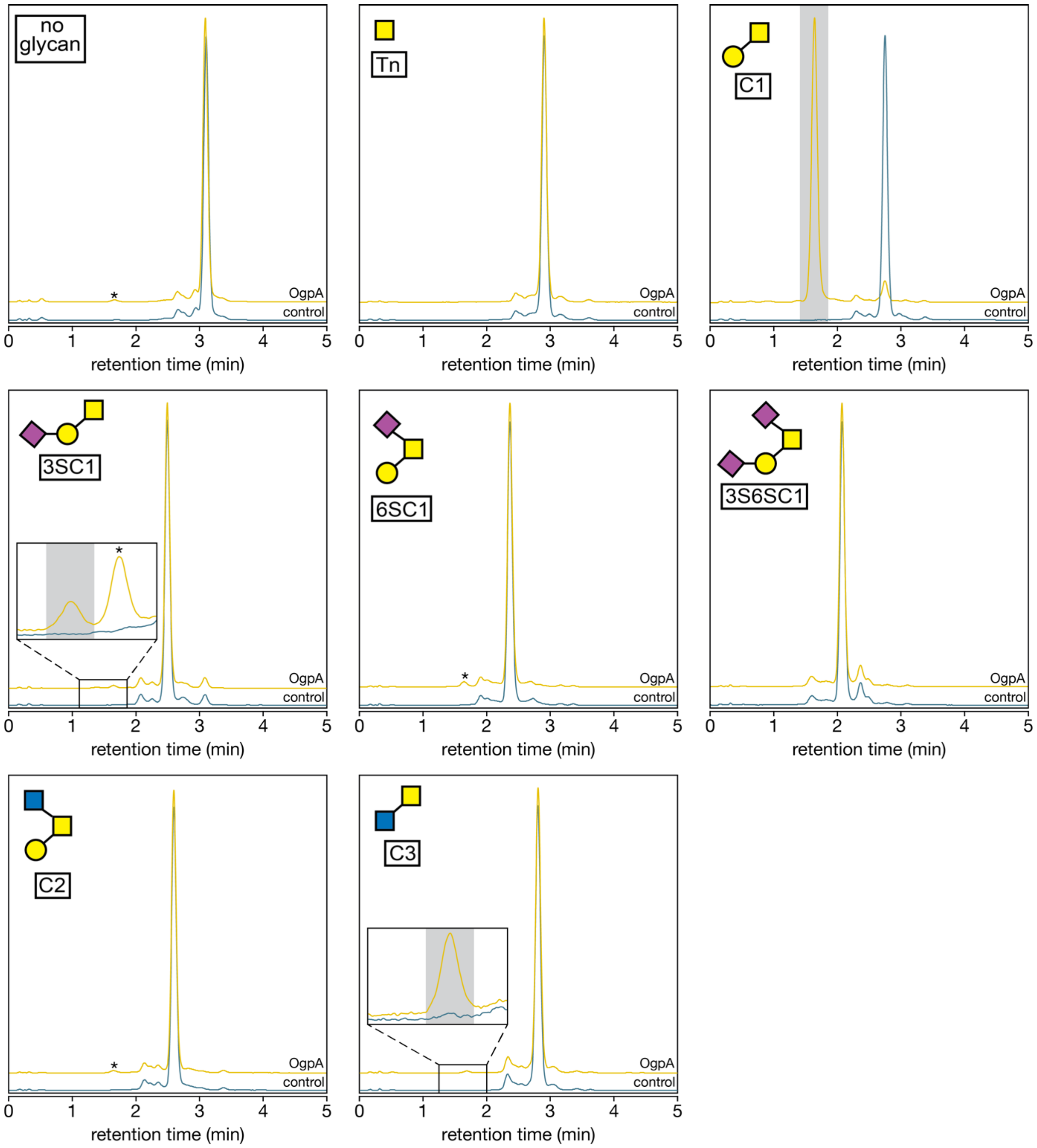

Supplementary Figure 8 | Substrate specificity of OgpA for *O*-glycopeptides. The different glycopeptides were incubated with OgpA overnight (18 h) and the reaction mixture was analyzed by high pH reverse phase HPLC with fluorecence detection. Each panel shows an overlay of an OgpA digestion (yellow) with a control reaction without the enzyme (teal). Reaction products are highlighted in grey. The asterisk marks peaks corresponding to digested C1 peptide. These originated from incomplete turnover during synthesis of the other core structures using C1 as starting material.

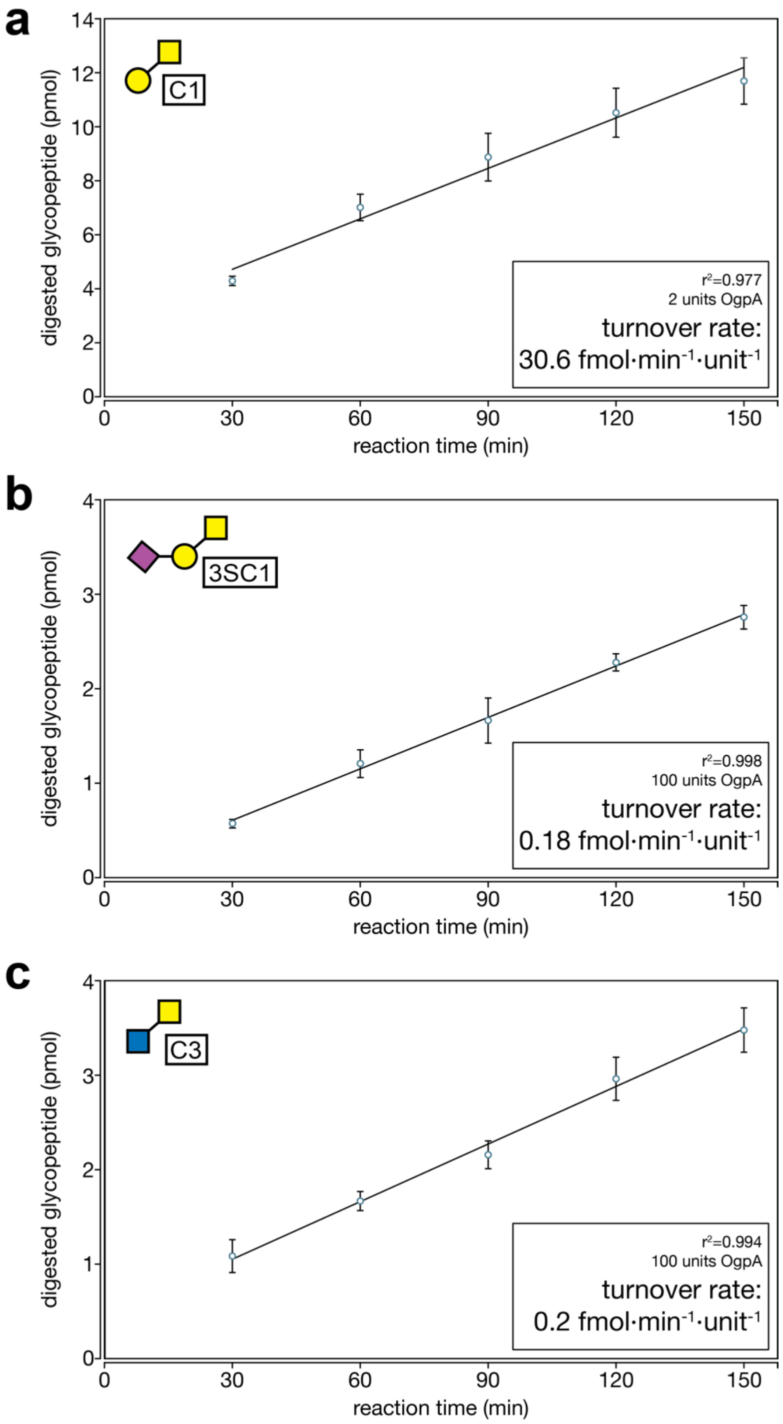

Supplementary Figure 9 | Substrate specificity of OgpA for *O*-glycopeptides. Glycopeptides were incubated with the indicated amounts of OgpA and the reaction products analyzed by reverse phase HPLC. Initial turnover rates were determined from reverse phase HPLC data of triplicate reactions using linear regression. Error bars represent the standard deviation of the three replicates.

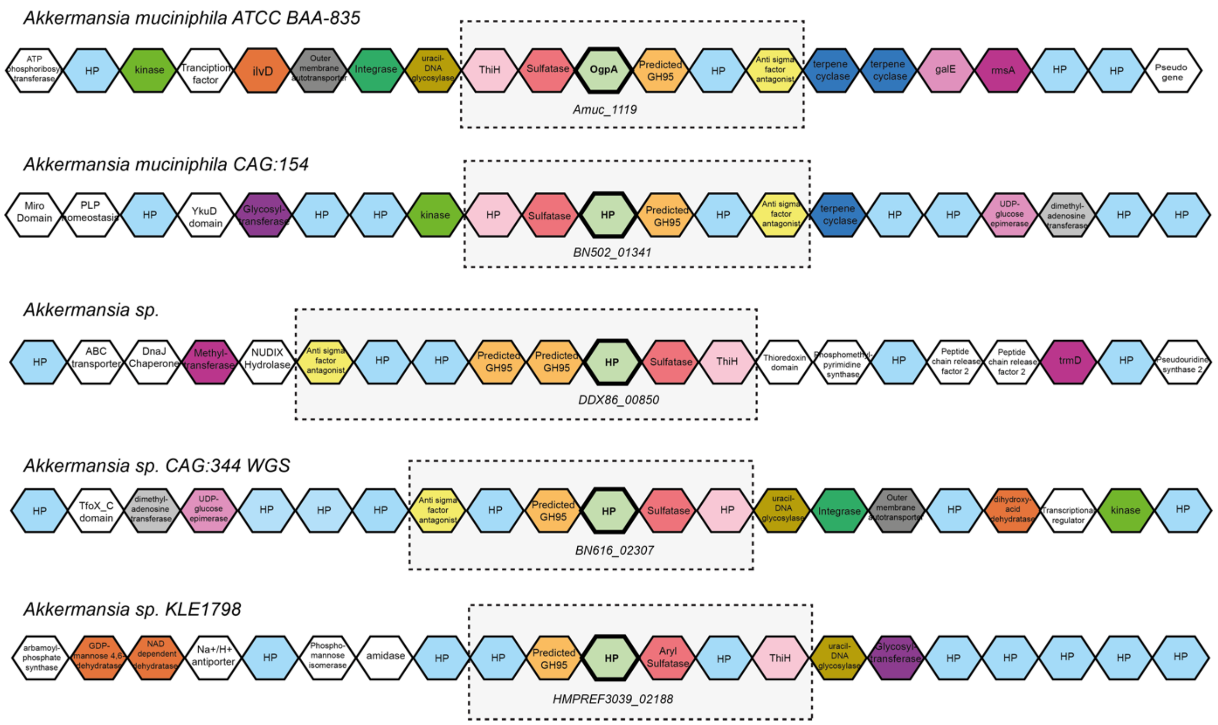

Supplementary Figure 10 | Genomic organization of *ogpA* gene in selected *A. muciniphila* strains. Linear distribution of 10 genes represented with hexagons upstream and downstream from *ogpA* gene (*Amuc_1119*) in *A. muciniphila ATCC BAA-835* strain in the first row. Same genomic representation of *ogpA* gene homologues in *CAG:154, sp. (DDX86_00850), CAG:344 WGS (BN616_02307)* and *KLE1798 (HMPREF3039_02188)* strains respectively in the following rows according to The National Center for Biotechnology Information (NCBI) Gene and Genome databases (https://www.ncbi.nlm.nih.gov). Similar functionality genes are highlighted in colors. *ogpA* gene and its homologues are highlighted in light green in the middle of the linear genomic representation. HP, hypothetical protein, refers to those genes that are predicted to encode an unknown function protein. In light grey dashed line boxes are indicated the genes close to *ogpA* gene and to its homologues which encode conserved functionality proteins in the compared *A. muciniphila* strains: HP in light blue, a predicted glycoside hydrolase (GH95) in orange, a sulphatase in red, o*gpA* gene and its homologues in light green, anti-sigma factor encoding gene in yellow and a dihydroxy-acid dehydratase related to thiamin and biotin synthesis encoding protein gene in light pink.

4. SUPPLEMENTARY REFERENCES

1. Abdullah, K. M., Udoh, E. A., Shewen, P. E. & Mellorsl, A. A neutral glycoprotease of Pasteurella haemolytica A1 specifically cleaves O-sialoglycoproteins. *Infect. Immun.* **60**, 56–62 (1992).

2. Malaker, S. A. *et al.* The mucin-selective protease StcE enables molecular and functional analysis of human cancer-associated mucins. *Proc. Natl. Acad. Sci. U. S. A.* **116**, 7278–7287 (2019).

3. Yu, A. C. Y., Worrall, L. J. & Strynadka, N. C. J. Structural insight into the bacterial mucinase StcE essential to adhesion and immune evasion during enterohemorrhagic *E. coli* infection. *Structure* **20**, 707–717 (2012).

4. Wang, P. & Granado, R. R. An intestinal mucin is the target substrate for a baculovirus enhancin. *Proc. Natl. Acad. Sci. U. S. A.* **94**, 6977–6982 (1997).

5. Ayala-Lujan, J. L. *et al.* Broad spectrum activity of a lectin-like bacterial serine protease family on human leukocytes. *PLoS One* **9**, e107920 (2014).

6. Noach, I. *et al.* Recognition of protein-linked glycans as a determinant of peptidase activity. *Proc. Natl. Acad. Sci. U. S. A.* **114**, E679–E688 (2017).

7. Nesta, B. *et al.* SslE elicits functional antibodies that impair in vitro mucinase activity and in vivo colonization by both intestinal and extraintestinal *Escherichia coli* strains. *PLoS Pathog.* **10**, e1004124 (2014).

8. Rawlings, N. D. *et al.* The MEROPS database of proteolytic enzymes, their substrates and inhibitors in 2017 and a comparison with peptidases in the PANTHER database. *Nucleic Acids Res.* **46**, D624–D632 (2018).

9. Auld, D. S. Catalytic mechanisms for metallopeptidases. in *Handbook of Proteolytic Enzymes* (eds. Rawlings, N. D. & Salvessen G.) 370–395 (Oxford: Academic Press, 2013).

10. Matthews, B. W. Structural basis of the action of thermolysin and related zinc peptidases. *Acc. Chem. Res.* **21**, 333–340 (1988).

11. Vallee, B. L. & Auld, D. S. Active-site zinc ligands and activated H_2O of zinc enzymes. *Proc. Natl. Acad. Sci. U. S. A.* **87**, 220–224 (1990).

12. Pelmenschikov, V. & Siegbahn, P. E. M. Catalytic mechanism of matrix metalloproteinases: two-layered ONIOM study. *Inorg. Chem.* **41**, 5659-66 (2002).

13. Bertini, I. *et al.* Snapshots of the reaction mechanism of matrix metalloproteinases. *Angew. Chemie - Int. Ed.* **46**, 7952–7955 (2006).

14. W. Matthews, B. Structural basis of the action of thermolysin and related zinc peptidases. *Acc. Chem. Res.* **21**, 333–340 (2002).

15. Pelmenschikov, V. & Siegbahn, P. E. M. Catalytic mechanism of matrix metalloproteinases: Two-layered ONIOM study. *Inorg. Chem.* **41**, 5659–5666 (2002).

16. Cerdà-Costa, N. & Gomis-Rüth, F. X. Architecture and function of metallopeptidase catalytic domains. *Protein Sci.* **23**, 123–144 (2014).

17. Dudev, T. & Lim, C. Tetrahedral vs octahedral zinc complexes with ligands of biological interest: A DFT/CDM study. *J. Am. Chem. Soc.* **122**, 11146–11153 (2000).
