# Supplementary material for: Structural basis of mammalian mucin processing by the human gut O-glycopeptidase OgpA from Akkermansia muciniphila
Source: Nat Commun. 2020 Sep 24;11:4844. doi: 10.1038/s41467-020-18696-y (PMC7518263; doi:10.1038/s41467-020-18696-y)
Supplement: Supplementary file 3 — Reporting Summary [file 41467_2020_18696_MOESM3_ESM.pdf]

nature research

Corresponding author(s): Marcelo E. Guerin
Jonathan Sjögren

Last updated by author(s): Aug 6, 2020

Reporting Summary

Nature Research wishes to improve the reproducibility of the work that we publish. This form provides structure for consistency and transparency in reporting. For further information on Nature Research policies, see our Editorial Policies and the Editorial Policy Checklist.

Statistics

For all statistical analyses, confirm that the following items are present in the figure legend, table legend, main text, or Methods section.

n/a	Confirmed	
☐	☒	The exact sample size (*n*) for each experimental group/condition, given as a discrete number and unit of measurement
☐	☒	A statement on whether measurements were taken from distinct samples or whether the same sample was measured repeatedly
☒	☐	The statistical test(s) used AND whether they are one- or two-sided<br>*Only common tests should be described solely by name; describe more complex techniques in the Methods section.*
☒	☐	A description of all covariates tested
☒	☐	A description of any assumptions or corrections, such as tests of normality and adjustment for multiple comparisons
☐	☒	A full description of the statistical parameters including central tendency (e.g. means) or other basic estimates (e.g. regression coefficient) AND variation (e.g. standard deviation) or associated estimates of uncertainty (e.g. confidence intervals)
☒	☐	For null hypothesis testing, the test statistic (e.g. *F*, *t*, *r*) with confidence intervals, effect sizes, degrees of freedom and *P* value noted<br>*Give P values as exact values whenever suitable.*
☒	☐	For Bayesian analysis, information on the choice of priors and Markov chain Monte Carlo settings
☒	☐	For hierarchical and complex designs, identification of the appropriate level for tests and full reporting of outcomes
☒	☐	Estimates of effect sizes (e.g. Cohen's *d*, Pearson's *r*), indicating how they were calculated

Our web collection on statistics for biologists contains articles on many of the points above.

Software and code

Policy information about availability of computer code

Data collection	Reverse phase HPLC on a Vanquish Duo UHPLC system equipped with an MSPac DS-10 desalting cartridge (both Thermo Fisher)
Data analysis	Vanquish Duo UHPLC system<br>xds (VERSION Jan 31, 2020)<br>ccp4 7.0<br>phenix-1.14-3260<br>coot 0.8.9.2<br>chimera 1.14<br>hollow 1.2<br>Autodock vina 1.1.2<br>Ligplot v.2.2

For manuscripts utilizing custom algorithms or software that are central to the research but not yet described in published literature, software must be made available to editors and reviewers. We strongly encourage code deposition in a community repository (e.g. GitHub). See the Nature Research guidelines for submitting code & software for further information.

Data

Policy information about availability of data

All manuscripts must include a data availability statement. This statement should provide the following information, where applicable:

- Accession codes, unique identifiers, or web links for publicly available datasets
- A list of figures that have associated raw data
- A description of any restrictions on data availability

The atomic coordinates and structure factors have been deposited with the Protein Data Bank, accession codes 6Z2D [http://dx.doi.org/10.2210/pdb6Z2D/pdb] (OgpAWT1), 6Z2O [http://dx.doi.org/10.2210/pdb6Z2O/pdb] (OgpAWT2), 6Z2P [http://dx.doi.org/10.2210/pdb6Z2P/pdb] (OgpAH205A/E206A-GD-SUB), and 6Z2Q [http://dx.doi.org/10.2210/pdb6Z2Q/pdb] (OgpAWT-GD-PRO). The following pdb accesion codes have been used for analysis in this manuscript: 3G42 [http://dx.doi.org/10.2210/pdb3G42/pdb], 1ATL [http://dx.doi.org/10.2210/pdb1ATL/pdb], 1ND1 [http://dx.doi.org/10.2210/pdb1ND1/pdb], 5JIP [http://dx.doi.org/10.2210/pdb5JIP/pdb], 6H7W [http://dx.doi.org/10.2210/pdb6H7W/pdb], 6BYI [http://dx.doi.org/10.2210/pdb6BYI/pdb], 5KD8 [http://dx.doi.org/10.2210/pdb5KD8/pdb], 5KDU [http://dx.doi.org/10.2210/pdb5KDU/pdb], 5KDX [http://dx.doi.org/10.2210/pdb5KDX/pdb], 3UJZ [http://dx.doi.org/10.2210/pdb3UJZ/pdb]. The protein sequences of OgpA from Akkermansia muciniphila (strain ATCC BAA-835 / Muc) [https://www.uniprot.org/uniprot/B2UR60], BN502_01341 from A. muciniphila CAG:154 [https://www.uniprot.org/uniprot/R6J0R4], DDX86_00850 from Akkermansia sp. [https://www.uniprot.org/uniprot/A0A354E6N0], BN616_02307 from Akkermansia sp. CAG:344 [https://www.uniprot.org/uniprot/R7DZB7], DF185_09660 from Marinifilum breve [https://www.uniprot.org/uniprot/A0A2V3ZZK2], BN783_01361 from Odoribacter sp. CAG:788 [https://www.uniprot.org/uniprot/R5PJX3], SAMN04488055_0630 from Chitinophaga niabensis [https://www.uniprot.org/uniprot/A0A1N6DD36], ECE50_27605 from Chitinophaga sp. Mgbs1 [https://www.uniprot.org/uniprot/A0A3S1CWX3], SAMN05660461_5684 from Chitinophaga ginsengisegetis [https://www.uniprot.org/uniprot/A0A1T5PAV1], ATE49_15630 from Elizabethkingia miricola [https://www.uniprot.org/uniprot/A0A1A6C9I9], BD94_1796 from Elizabethkingia anophelis NUHP1 [https://www.uniprot.org/uniprot/A0A077EDG2], IQ37_10980 from Chryseobacterium piperi [https://www.uniprot.org/uniprot/A0A086BCN5], P278_02110 from Zhouia amylolytica [https://www.uniprot.org/uniprot/W2URL6], W5A_07577 from Imtechella halotolerans [https://www.uniprot.org/uniprot/I0WF82] have been used for analysis in this manuscript and have been obtained from the Uniprot database. All other data that support the findings of this study are available from the corresponding authors on reasonable request.

Field-specific reporting

Please select the one below that is the best fit for your research. If you are not sure, read the appropriate sections before making your selection.

☒ Life sciences ☐ Behavioural & social sciences ☐ Ecological, evolutionary & environmental sciences

For a reference copy of the document with all sections, see nature.com/documents/nr-reporting-summary-flat.pdf

Life sciences study design

All studies must disclose on these points even when the disclosure is negative.

Sample size	No sample size calculation was performed. Hydrolytic/Kinetic experiments were performed in triplicates.
Data exclusions	No data were excluded from the hydrolytic/kinetic experiments.
Replication	Initial turnover rates were determined from reverse phase HPLC data of triplicate reactions using linear regression (please see Methods section and Supplementary Figure 9).
Randomization	N/A
Blinding	N/A

Reporting for specific materials, systems and methods

We require information from authors about some types of materials, experimental systems and methods used in many studies. Here, indicate whether each material, system or method listed is relevant to your study. If you are not sure if a list item applies to your research, read the appropriate section before selecting a response.

Materials & experimental systems

n/a	Involved in the study	
☒	☐	Antibodies
☒	☐	Eukaryotic cell lines
☒	☐	Palaeontology and archaeology
☒	☐	Animals and other organisms
☒	☐	Human research participants
☒	☐	Clinical data
☒	☐	Dual use research of concern

Methods

n/a	Involved in the study	
☒	☐	ChIP-seq
☒	☐	Flow cytometry
☒	☐	MRI-based neuroimaging
